# Tumor-Shed Antigen Affects Antibody Tumor Targeting: Comparison of Two ^89^Zr-Labeled Antibodies Directed against Shed or Nonshed Antigens

**DOI:** 10.1155/2018/2461257

**Published:** 2018-03-12

**Authors:** Jae-Ho Lee, Heejung Kim, Zhengsheng Yao, Lawrence P. Szajek, Luigi Grasso, Insook Kim, Chang H. Paik

**Affiliations:** ^1^Radiopharmaceutical Laboratory, Nuclear Medicine, Radiology and Imaging Sciences, Clinical Center, NIH, Bethesda, MD, USA; ^2^Positron Emission Tomography Department, Clinical Center, NIH, Bethesda, MD, USA; ^3^Morphotek, Inc., Exton, PA, USA; ^4^Applied/Developmental Research Directorate, Leidos Biomedical Research, Inc., Frederick National Laboratory for Cancer Research, Frederick, MD, USA

## Abstract

We investigated the effect of shed antigen mesothelin on the tumor uptake of amatuximab, a therapeutic anti-mesothelin mAb clinically tested in mesothelioma patients. The B3 mAb targeting a nonshed antigen was also analyzed for comparison. The mouse model implanted with A431/H9 tumor, which expresses both shed mesothelin and nonshed Lewis-Y antigen, provided an ideal system to compare the biodistribution and PET imaging profiles of the two mAbs. Our study demonstrated that the tumor and organ uptakes of ^89^Zr-B3 were dose-independent when 3 doses, 2, 15, and 60 *μ*g B3, were compared at 24 h after injection. In contrast, tumor and organ uptakes of ^89^Zr-amatuximab were dose-dependent, whereby a high dose (60 *μ*g) was needed to achieve tumor targeting comparable to the low dose (2 *μ*g) of ^89^Zr-B3, suggesting that shed mesothelin may affect amatuximab tumor targeting as well as serum half-life. The autoradiography analysis showed that the distribution of ^89^Zr-B3 was nonuniform with the radioactivity primarily localized at the tumor periphery independent of the B3 dose. However, the autoradiography analysis for ^89^Zr-amatuximab showed dose-dependent distribution profiles of the radiolabel; at 10 *μ*g dose, the radiolabel penetrated toward the tumor core with its activity comparable to that at the tumor periphery, whereas at 60 *μ*g dose, the distribution profile became similar to those of ^89^Zr-B3. These results suggest that shed antigen in blood may act as a decoy requiring higher doses of mAb to improve serum half-life as well as tumor targeting. Systemic mAb concentration should be at a severalfold molar excess to the shed Ag in blood to overcome the hepatic processing of mAb-Ag complexes. On the other hand, mAb concentration should remain lower than the shed Ag concentration in the tumor ECS to maximize tumor penetration by passing binding site barriers.

## 1. Introduction

Monoclonal antibody- (mAb-) based solid tumor therapy is challenging due to various parameters that can impede the tumor delivery and penetration of mAb. Some parameters are related to tumor environment factors, including vascular and stroma density, interstitial pressure, and tumor binding site barriers [1–3]. In addition, the antigen- (Ag-) mediated tumor targeting of mAb may be hampered by the presence of high levels of shed Ag in blood which could act as a decoy preventing mAbs from binding to antigens expressed on tumor cells [4–7]. We previously reported that shed mesothelin (MSLN) in blood circulation negatively affected the tumor targeting of amatuximab labeled with ^111^In or ^64^Cu by increasing its liver and spleen uptakes while decreasing its blood retention and tumor uptake when the injection dose of amatuximab was not sufficient to saturate the shed MSLN in blood circulation [5, 6]. In contrast to the negative effect of shed MSLN in blood circulation, a mathematical simulation suggested that the shed MSLN in the extracellular space (ECS) could positively affect the tumor uptake by improving the penetration of the antibody toward tumor core [8].

In this study, we investigated the effects of shed Ag on the tumor targeting and penetration of mAb. To achieve this goal, we used a nude mouse model implanted with A431/H9 tumor that overexpresses both shed MSLN (5 million Ag molecules/cell) and nonshed Lewis-Y (4 million Ag molecules/cell). MSLN is a membrane glycoprotein of 40 kDa that is actively internalized into the cell's cytosol as well as shed from the tumor cell surface, generating soluble MSLN in the tumor's interstitial space and blood circulation with its concentrations proportional to the size of tumor [5, 7, 9]. Lewis-Y is a carbohydrate antigen that is not actively internalized nor shed from the tumor surface [9–13]. As model antibodies, we used two mAbs: anti-MSLN mAb amatuximab (mouse/human chimeric antibody with 82.6% amino acid sequence identity to a human IgG1*κ* and 10^−9^ M *K*_*D*_ binding affinity), a therapeutic mAb currently investigated in mesothelioma patients, and control anti-Lewis-Y mAb B3 (murine IgG1*κ* with 10^−8^ M *K*_*D*_ binding affinity). Comparative studies using this system enabled us to define the effects of shed Ag on the tumor uptake and penetration of mAb apart from the effects of other factors such as vascular density, high interstitial fluid pressure (IFP), and extracellular protein contents.

The two mAbs were labeled with ^89^Zr (decay half-life, 78.4 h), which decays with a low positron emission energy of 395.5 keV, allowing for PET imaging with higher resolution [14–16]. ^89^Zr can enhance PET radioimmune detection of labeled mAbs and extend autoradiography preparation time due to the longer half-life and higher imaging resolution compared with ^111^In and ^64^Cu. In addition to the biodistribution (BD), PET imaging, and autoradiography studies for the two ^89^Zr-labeled mAbs, here we also report a new autoradiography analysis method to define the tumor uptake profile of the two ^89^Zr mAbs irrespective of tumor size and shape.

## 2. Materials and Methods

Amatuximab was obtained from Morphotek, Inc. (Exton, PA), and B3 was provided by Dr. Ira Pastan (LMB, NCI, NIH). p-Isothiocyanatobenzyl-desferrioxamine (p-SCN-Df) was purchased from Macrocyclics, Inc. (Dallas, TX). Zirconium-89 (^89^Zr) was produced at the National Institute of Health (Bethesda, MD) cyclotron facility using a 16.5 MeV proton cyclotron (PET trace, General Electric, Fairfield, CT) by proton irradiation (beam energy; 14 MeV, current; 20 *µ*A) (*p*, *n*) reaction (2~5 h) on ^89^Y-metal mesh (200 mg, 4N purity, American Elements). ^89^Zr was separated as ^89^Zr-oxalate from irradiated ^89^Y-metal mesh using 0.1 M oxalic acid solution [16].

### 2.1. Conjugation of p-SCN-Df to Amatuximab or B3 Antibody

The mAbs were radiolabeled with ^89^Zr using desferrioxamine (Df) with an isothiocyanate linker as a chelating agent following a method of Vosjan et al. [17]. Briefly, amatuximab or B3 was reacted with p-SCN-Df at a molar ratio of 1 : 3 in 0.1 M sodium bicarbonate, at pH 9.5 at 37°C. The Df-amatuximab or Df-B3 conjugate was purified with a size exclusion PD-10 column (GE Healthcare Bio-Sciences AB, Uppsala, Sweden) and concentrated with a Microcon® filter with a 30 kDa cutoff (Millipore, Bedford, MA). The column or the filter was pretreated with 25 mg BSA containing 1 *µ*mol DTPA to block nonspecific protein binding sites and remove potential metal contaminants and then washed with metal-free sodium acetate (0.25 M, pH 5.5). The mAb concentrations were measured according to the method of Bradford [18]. The level of p-SCN-Df conjugated per mAb was determined by the percent ^89^Zr distribution between the peaks corresponding to Df-mAb and free DF on the size exclusion HPLC when the product mixture was radiolabeled as described below.

### 2.2. Radiolabeling

Purified Df-amatuximab or Df-B3 (1.0 mg/ml, 6.9 *µ*M) was labeled with ^89^Zr (344 MBq/0.15 M oxalic acid for Df-amatuximab and 148 MBq/0.15 M oxalic acid for Df-B3), which was neutralized with a solution containing sodium carbonate (0.135 M)/HEPES buffer (pH = 7, 0.25 M) and d-mannitol (5.5 mg/mL) at 25°C for 1 h. One mL of 0.25 M sodium acetate containing 5 mg/mL gentisic acid (pH 5.5) was then added to the reaction solution. The labeled product was purified with PD 10 columns eluted with metal-free elution buffer (0.25 M sodium acetate containing 5 mg/mL gentisic acid, pH 5.5). Each PD 10 column was pretreated with 25 mg BSA containing 1 *µ*mol DTPA to block nonspecific protein binding sites and remove potential metal contaminants and then washed with metal-free elution buffer. The radiolabeling yield and the radiochemical purity were assessed by analytical size exclusion HPLC (Gilson, Middleton, WI) before and after the purification (please see the Materials and Methods section in the previous paper for detailed information [5]). The radiolabeling yield was determined based on the distribution of ^89^Zr between ^89^Zr-labeled amatuximab (retention time: 8.53 min) and unbound ^89^Zr (retention time: 9.45 min) on the HPLC profiles.

### 2.3. Immunoreactivity Determination

The immunoreactivity of ^**89**^Zr-amatuximab or B3 was determined by a modified cell-binding assay of Lindmo and Bunn [19], as previously reported [5]. Aliquots (5 ng/50 *µ*L) of the conjugate samples were incubated side-by-side with an increasing number of A431/H9 cells (positive for both mesothelin and Lewis-Y; 2 × 10^4^–1 × 10^6^ cells) in 100 *µ*L of PBS with 1% BSA at 4°C for 3 hours. Nonspecific binding to the cells was determined by performing the cell-binding assays under a condition of excess amount of antibodies (50 *µ*g amatuximab and 50 *µ*g B3).

### 2.4. Tumor Model

A431/H9 is a derivative of the A431 epidermoid carcinoma cell line that has been stably transfected with vectors for human mesothelin and grown in media supplemented with 750 mg/mL G-418 (Geneticin) for selection [20]. This cell line was grown at 37°C with 5% CO_2_ in media supplemented with 10% FBS, 2 mmol/L L-glutamine, 100 U penicillin, and 100 mg streptomycin (Invitrogen Corporation). The cell line was authenticated at the source and grown from frozen stocks prepared from an early passage of the original line [21].

### 2.5. Biodistribution Studies

For the BD studies with ^89^Zr-labeled amatuximab or B3 conjugate with Df molecules, groups (*n* = 4-5 mice/group) of mice were injected (i.v.) with ^89^Zr-labeled mAb conjugates (111 kBq for^ 89^Zr-amatuximab; 74 kBq for ^89^Zr-B3) mixed with corresponding unlabeled intact antibodies (2, 10, or 60 *µ*g amatuximab; 2, 15, or 60 *µ*g B3) in 0.2 mL PBS containing 1% BSA. The tumor sizes at the time of the BD studies were as follows: 245.5 ± 6.5, 190.8 ± 5.0, and 226.4 ± 7.1 mm^3^ for 2, 10, and 60 *µ*g amatuximab, respectively, and 205.9 ± 3.4, 321.2 ± 9.0, and 372.5 ± 8.2 mm^3^ for 2, 15, and 60 *µ*g B3, respectively. The animals were euthanized at 24 hours by CO_2_ inhalation and exsanguination by cardiac puncture. We performed the BD studies as described previously (please see the Materials and Methods section in the previous paper for detailed information [5]). All animal experiments were performed under a protocol approved by the NIH Animal Care and Use Committee.

### 2.6. PET Imaging

PET imaging studies were performed as described in the previous study of ^64^Cu-NOTA-amatuximab (please see PET Imaging section in the previous paper [5]). The mice (*n* = 5) with A431/H9 tumor were injected (i.v.) with ^89^Zr-amatuximab (2.96 MBq/10 or 60 *µ*g total amatuximab) or ^89^Zr-B3 (2.22 MBq/15 or 60 *µ*g total B3) in 0.2 ml of normal saline through the tail vein and then 15 min static PET scans were performed at 3, 24, and 48 h p.i. The tumor sizes at the time of the PET imaging were as follows: 429 ± 141 mm^3^ (range: 253–599 mm^3^) for 10 *µ*g amatuximab and 406 ± 23 mm^3^ (range: 385–440 mm^3^) for 60 *µ*g amatuximab; 700 ± 220 mm^3^ (range: 436–1042 mm^3^) for 15 *µ*g B3 and 441 ± 126 mm^3^ (range: 304–630 mm^3^) for 60 *µ*g B3.

### 2.7. Autoradiography and Its Analysis

**Figure 1:**
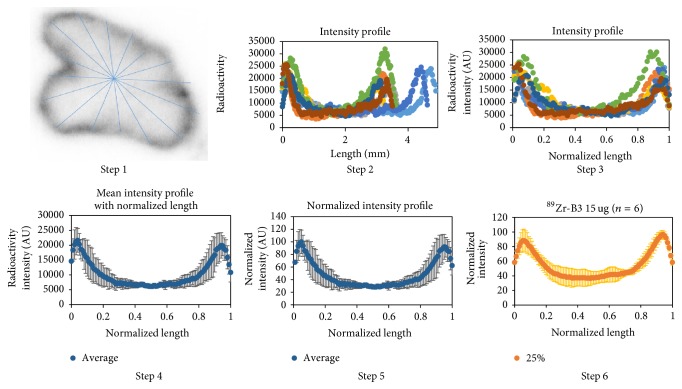
Procedure of a new normalized length analysis method for autoradiography. Step 1: draw lines on autoradiography image imported into ImageJ. Step 2: export each line plot data file and plot the original intensity versus line length profile. Step 3: normalize each length and replot with the interpolation function, interp1, in Matlab. Step 4: plot mean and standard deviation intensity profile with normalized length. Step 5: plot normalized intensity profile with normalized length. Step 6: repeat steps 1–5 for each tumor section and get mean normalized intensity profile to represent tumor sections cut at 25%, 50%, and 75% regions of the long tumor axis.

For Ex vivo autoradiography, the mice were selected according to tumor volume by PET and euthanized immediately after 48 h PET imaging session and the tumors were excised. The tumors with the following sizes were used for autoradiography studies: 285 ± 46 mm^3^ (range: 252–318 mm^3^; *n* = 2) and 388 ± 5 mm^3^ (range: 385–392 mm^3^; *n* = 2) for 10 and 60 *µ*g amatuximab, respectively, and 574 ± 121 mm^3^ (range: 437–666 mm^3^; *n* = 3) and 364 ± 60 mm^3^ (range: 304–424 mm^3^; *n* = 3) for 15 and 60 *µ*g B3, respectively. The tumors were embedded and frozen in Tissue-Tek® CRYO-OCT compound (Sakura® Finetek USA Inc., Torrance, CA, USA) at −20°C for 3 h. Serial 20 *µ*m thick short axis sections were cut in 400 *µ*m intervals covering the entire tumor. Two or three consecutive tumor slices were selected at 3 tumor regions (25%, 50%, and 75% long axis regions from the tumor surface) as representative sections throughout the tumor and exposed on the phosphor screen for 16 h. Signals were obtained by the use of the Typhoon FLA 7000 (GE Healthcare Life Sciences, Pittsburgh, PA, USA) with 25 *µ*m pixel resolution and analyzed with Image Quant TL8.1 software. Values were grouped together from the 3 tumor regions to represent a tumor. Each tumor was treated as an independent sample. To analyze the microdistribution of the radioactivity in the tumor sections, we introduced a normalized length analysis method as described below. The first line was drawn along the longest axis, and the second line was drawn along a short axis perpendicularly at the center of the first longest line (see Figure 1). The center was selected as the point where the two lines meet. Additional lines were drawn evenly and continuously between the two original lines passing through the same center point (total of 8 lines). Radioactivity profile of each line was analyzed with ImageJ (NIH, Bethesda, MD) and exported into Excel files to redefine values with Matlab's interpolation function interp1. The maximum length of each line in *x*-axis was normalized to 1 to correct for the differences in the length of each line for reconstruction of the radioactivity versus tumor penetration distance profiles of each tumor section. The maximum signal intensity within each tumor section in *y*-axis was also normalized to 100 to correct for the differences in the signal intensity between each tumor section. Mean radioactivity versus distance profiles with standard deviation were then reconstructed for tumor sections obtained at 25%, 50%, and 75% regions.

### 2.8. Statistical Analysis

Statistical analysis was performed using ANOVA for comparing multiple groups, and Student's *t*-test was performed for unpaired data between two groups. All tests were two-sided, and a probability value (*p*) of less than 0.05 was considered significant.

## 3. Results

### 3.1. Characterization of ^89^Zr mAbs

The level of Df conjugation was 1.6 ± 0.3 (*n* = 3) for B3 and 0.9 ± 0.2 (*n* = 3) for amatuximab. The ^89^Zr-labeled mAbs were purified on PD-10 columns eluted with acetate buffer (pH 5.5) containing gentisic acid at 5 mg/ml. The purified products were >95% radiochemically pure based on the size exclusion HPLC profiles. The specific activities of the purified product were 296 kBq/*µ*g for ^89^Zr-amatuximab and 148 kBq/*µ*g for ^89^Zr-B3. The immunoreactivities of  ^89^Zr-amatuximab and ^89^Zr-B3 were 84.0 ± 2.2% (*n* = 2) and 70.0 ± 1.0% (*n* = 2), respectively.

### 3.2. Biodistribution Studies

**Figure 2:**
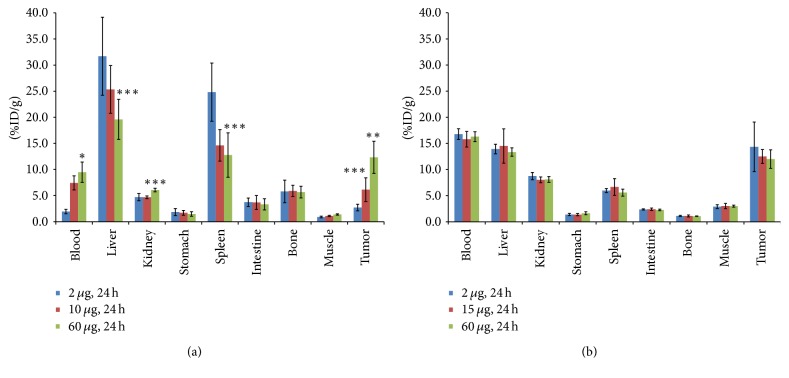
Effects of total injection dose of mAb on the BD of ^89^Zr-mAb in nude mice (*n* = 4-5 per group) with A431/H9 tumor: (a) the BD data from ^89^Zr-amatuximab (111 kBq) with different injection doses of amatuximab (2 *µ*g, 10 *µ*g, and 60 *µ*g) are compared at 24 h p.i. in nude mice with tumor sizes 245.5 ± 6.5, 190.8 ± 5.0, and 226.4 ± 7.1 mm^3^ for 2, 10, and 60 *µ*g amatuximab. The results demonstrate that tumor uptake and blood retention significantly increased whereas liver uptake decreased as the antibody dose was increased; (b) the BD data from ^89^Zr-B3 (74 kBq) with different injection doses of B3 (2 *µ*g, 15 *µ*g, and 60 *µ*g) are compared at 24 h p.i. in nude mice with tumor sizes 205.9 ± 3.4, 321.2 ± 9.0, and 372.5 ± 8.2 mm^3^ for 2, 15, and 60 *µ*g. The results demonstrate that tumor uptake, blood retention, and liver uptake were not affected by the antibody dose. The data are mean ± SD. Analysis of statistical significance in each organ uptake data compared to the data from 2 *µ*g injection: ^*∗*^*p* < 0.001, 0.001 < ^*∗∗*^*p* < 0.01, and 0.01 < ^*∗∗∗*^*p* < 0.05; column: mean; bar: SD.

The results of comparative BD studies at 24 h indicated that the uptake of ^89^Zr-amatuximab in tumor, liver, spleen, and blood directly correlated with dose levels whereas the uptake of anti-Lewis-Y antibody ^89^Zr-B3 in these organs was dose-independent. In fact, ^89^Zr-amatuximab tumor uptake and blood retention increased as the injection dose increased (Figure 2(a) and Table 1). However, the liver and spleen uptake decreased as the injection dose increased. The tumor-to-organ ratios increased and conversely the tumor-to-blood ratio decreased as the dose increased, as previously reported for ^64^Cu-NOTA-amatuximab [5]. In contrast, a dose effect on tumor uptake, blood retention, and liver uptake, as well as the tumor-to-organ and the tumor-to-blood ratios for ^89^Zr-B3, was not appreciable (Figure 2(b) and Table 1).

### 3.3. PET Imaging Studies

**Figure 3:**
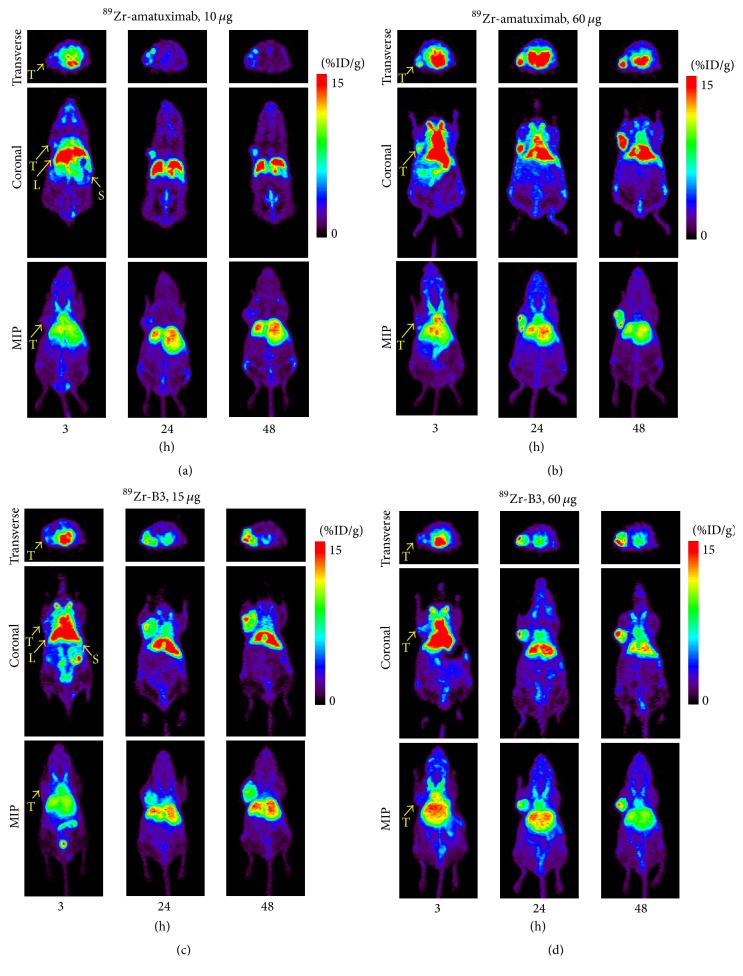
Representative PET images of ^89^Zr-mAb in nude mice with A431/H9 tumor: (a) ^89^Zr-amatuximab (2.96 MBq) coinjected with 10 *µ*g and 60 *µ*g amatuximab; (b) ^89^Zr-B3 (2.22 MBq) coinjected with 15 *µ*g and 60 *µ*g B3. Fifteen-minute static PET scans were performed at 3, 24, and 48 h p.i. PET images demonstrate that (a) the tumor uptake significantly increased when the injection dose of ^89^Zr-amatuximab was 60 *µ*g compared with 10 *µ*g dose and (b) there was no significant dose effect on the tumor uptake of ^89^Zr-B3 and the tumor uptake gradually increased over time.

**Figure 4:**
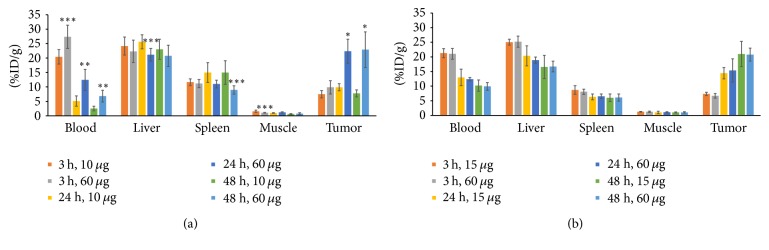
Effect of mAb dose on accumulation of ^89^Zr-mAb in nude mice (*n* = 5 per group) with A431/H9 tumor by PET analysis: (a) effect of amatuximab dose (10 *µ*g and 60 *µ*g) on accumulation of ^89^Zr-amatuximab (2.96 MBq) in nude mice with tumor (429 ± 141 mm^3^; range: 253–599 mm^3^) for 10 *µ*g amatuximab and (406 ± 23 mm^3^; range: 385–440 mm^3^) for 60 *µ*g amatuximab. (b) Effect of B3 antibody dose (15 *µ*g and 60 *µ*g) on accumulation of ^89^Zr-B3 (2.22 MBq) in nude mice with tumor (700 ± 220 mm^3^; range: 436–1042 mm^3^) for 15 *µ*g B3 and (441 ± 126 mm^3^; range: 304–630 mm^3^) for 60 *µ*g B3. The uptake value (%ID/g) was calculated by ROI analysis of PET images. Analysis of statistical significance in each organ uptake data (a) compared to the data from 10 *µ*g for amatuximab or (b) 15 *µ*g for B3. ^*∗*^*p* < 0.001, 0.001 < ^*∗∗*^*p* < 0.01, and 0.01 < ^*∗∗∗*^*p* < 0.05.

The findings from the BD studies were supported by the PET imaging results. ^89^Zr-amatuximab tumor uptake was visualized as early as 3 h postinjection (p.i.) at both dose levels, while the majority of radioactivity was still localized in heart (blood pool) and liver (Figure 3(a)). During the 3–48 h period, ^89^Zr-amatuximab was cleared rapidly from blood at the 10 *µ*g dose while tumor uptake remained unchanged (Figures 3(a) and 4(a)). In contrast, at the 60 *µ*g dose ^89^Zr-amatuximab cleared more gradually from blood and produced a drastic increase in tumor uptake (%ID/g) at 24 h and 48 h (Figure 4). The tumor uptake (%ID/g) was 7.46 ± 1.26, 9.88 ± 1.25, and 7.74 ± 1.22 at 3, 24, and 48 h, respectively, for 10 *µ*g and 9.85 ± 21.3, 22.37 ± 4.18, and 22.90 ± 6.18 at 3, 24, and 48 h, respectively, for 60 *µ*g. Blood retention (%ID/g) was 20.43 ± 2.49, 5.09 ± 1.78, and 2.52 ± 0.78 at 3, 24, and 48 h, respectively, for 10 *µ*g and 27.37 ± 4.03, 12.44 ± 3.66, and 6.80 ± 2.04 at 3, 24, and 48, respectively, for 60 *µ*g. Liver uptake (%ID/g) was 24.17 ± 3.11, 25.64 ± 2.43, and 23.03 ± 3.51 at 3, 24, and 48 h, respectively, for 10 *µ*g and 22.33 ± 3.89, 21.14 ± 2.21, and 20.78 ± 3.67 at 3, 24, and 48 h, respectively, for 60 *µ*g. Thus, these data indicate that a higher injection dose of amatuximab is advantageous for the tumor visualization by PET. The tumor-to-organ and the tumor-to-blood ratios from PET imaging had similar values observed in ^64^Cu-NOTA-amatuximab study [5]. The tumor-to-liver ratio showed a value >1 at 24 and 48 h p.i. for 60 *µ*g but a value <1 for 10 *µ*g (Table 2), suggesting that it is feasible to visualize tumors in the upper abdomen with the higher dose.

The PET images of ^89^Zr-B3 showed tumor uptake as early as 3 h p.i. at both 15 and 60 *µ*g doses. Compared to ^89^Zr-amatuximab, the PET images from ^89^Zr-B3 did not show any significant dose effects on its uptake and the clearance pharmacokinetics from tumor, blood, and liver (Figure 3(b)). The tumor uptake of the ^89^Zr-B3 increased steadily over a 48 h period while clearing gradually from the blood and the liver as follows (Figure 4): tumor uptake (%ID/g) of 7.36 ± 0.54, 14.43 ± 1.94, and 20.99 ± 4.33 at 3, 24, and 48 h, respectively, for 15 *µ*g and 6.71 ± 0.78, 15.36 ± 3.98, and 20.78 ± 2.21 at 3, 24, and 48 h, respectively, for 60 *µ*g; blood retention (%ID/g) of 21.28 ± 1.54, 12.99 ± 2.83, and 10.21 ± 1.92 at 3, 24, and 48 h, respectively, for 15 *µ*g and 21.05 ± 1.85, 12.40 ± 0.56, and 9.92 ± 1.33 at 3, 24, and 48 h, respectively, for 60 *µ*g; liver uptake (%ID/g) of 25.04 ± 1.03, 20.37 ± 3.4, and 16.55 ± 3.98 at 3, 24, and 48 h, respectively, for 15 *µ*g and 25.21 ± 1.90%ID/g, 18.90 ± 1.06, and 16.72 ± 1.83 at 3, 24, and 48 h, respectively, for 60 *µ*g. The tumor-to-organ and the tumor-to-blood ratios from PET imaging did not change as the dose increased (Table 2).

These results suggest a significant difference in biodistribution characteristics between ^89^Zr-amatuximab and ^89^Zr-B3, which may be due to the presence of shed mesothelin in blood and tumor tissue that may affect the serum half-life as well as Ag-specific tumor uptake of radiolabeled amatuximab as previously reported [5].

### 3.4. Autoradiography Studies

**Figure 5:**
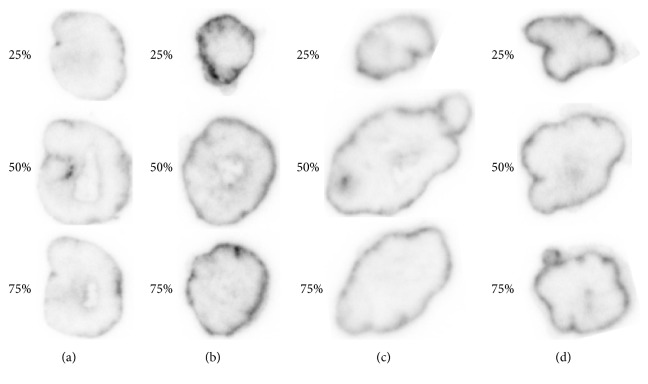
Representative autoradiography images of ^89^Zr-labeled antibody. ^89^Zr-amatuximab (2.96 MBq) coinjected with (a) 10 *µ*g and (b) 60 *µ*g amatuximab; ^89^Zr-B3 (2.22 MBq) coinjected with (c) 15 *µ*g and (d) 60 *µ*g B3 mAb.

**Figure 6:**
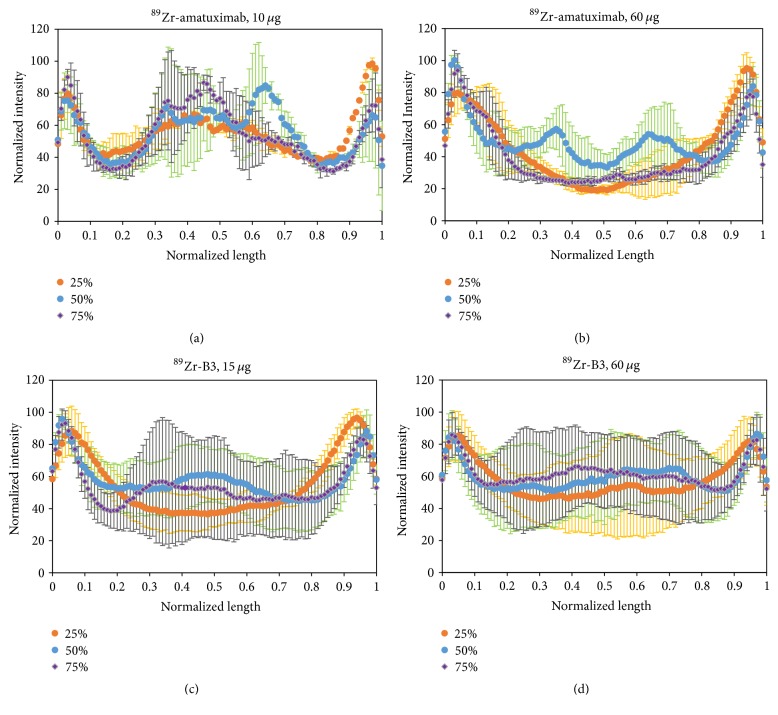
Autoradiography analysis of ^89^Zr-mAbs depicted as normalized intensity versus normalized length profiles: ^89^Zr-amatuximab (2.96 MBq) was coinjected with (a) 10 *µ*g and (b) 60 *µ*g amatuximab. ^89^Zr-B3 (2.22 MBq) was coinjected with (c) 15 *µ*g and (d) 60 *µ*g B3. The results demonstrate that the normalized radioactivity of ^89^Zr-amatuximab at the tumor core and the periphery was similar with the low dose of amatuximab but the relative ^89^Zr intensity at the tumor periphery was higher as the injection dose was increased to 60 *µ*g. In contrast, the radioactivity profile of ^89^Zr-B3 was not affected by the B3 dose between 15 and 60 *µ*g B3 and the radioactivity preferentially accumulated in the tumor periphery.

To investigate if the shed mesothelin in the ECS of tumor could affect the penetration of ^89^Zr-amatuximab, we performed the autoradiography of tumor segments at 48 h p.i. immediately after the completion of the PET imaging studies. The data of autoradiography images analysis (see Figure 1 for image analysis method) demonstrated that ^89^Zr-amatuximab had similar radioactivity intensity at the tumor core and the periphery when the injection dose was 10 *µ*g (Figures 5(a) and 6(a)). However, increasing the dose to 60 *µ*g increased the uptake to the periphery but not to the center of the tumor (Figures 5(b) and 6(b)). In contrast, the radioactivity data and the normalized radioactivity versus distance profiles for ^89^Zr-B3 showed that the uptake peaked at the tumor periphery while it decreased rapidly toward the tumor core at either dose levels (Figures 5(c), 5(d), 6(c), and 6(d)).

## 4. Discussion

The studies reported here highlight an important property of anti-mesothelin mAb amatuximab as compared to anti-Lewis-Y mAb B3 for the Ag-mediated targeting of A431/H9 tumor overexpressing both a shed Ag, mesothelin, and a nonshed Ag, Lewis-Y, in a mouse model. The comparative BD, PET imaging, and autoradiography studies between these two mAbs allowed us to define the effect of shed Ag on the tumor targeting and penetration, apart from the effects of other factors related to the tumor microenvironment. While the biodistribution and tumor uptake of ^89^Zr-B3 were not significantly affected by the injected dose, the tumor uptake of ^89^Zr-amatuximab increased whereas the uptake in liver and spleen decreased as the injected dose increased. This finding is consistent with the previously reported dose-dependent effect on the biodistribution and tumor uptake of ^64^Cu-NOTA-amatuximab [5]. The BD and tumor uptake of ^89^Zr-B3 were not appreciably affected by the injection dose levels because its target, Lewis-Y Ag, is not shed by tumor cells. The ROI analysis of PET images corresponded to the BD results and is consistent with the hypothesis of a decoy effect caused by soluble shed Ag in blood and tumor.

These findings could be explained by a threshold effect whereby ^89^Zr-amatuximab is mostly bound to shed MSLN in blood at the lower dose (10 *µ*g) and sequestered into the reticular endothelial system of liver and spleen, thereby lowering both blood retention and tumor uptake. Based on this knowledge, we hypothesized that a dose of 60 *µ*g could be sufficiently high to saturate shed MSLN as well as create an excess of ^89^Zr-amatuximab available for a more effective tumor targeting. This effect could be explained by correlating the concentration of shed MSLN in the blood and in the ECS of A431/H9 tumor, as estimated in previous studies [8, 22, 23], similarly to that of ^64^Cu-NOTA-amatuximab. ^89^Zr-amatuximab injected at 10 *µ*g dose results in an estimated blood concentration of 43.3 nM (assuming blood volume of 1.6 mL for a 20 g mouse) immediately after injection and 1.75 nM at 48 h (based on 2.52%ID/g blood in Figure 3). Because the average concentration of shed MSLN in the blood is ~6 nM in a tumor of ~300 mm^3^, ^89^Zr-amatuximab (1.75 nM) would be mostly bound to shed MSLN in blood during a 48 h period and this complex sequestered into liver and spleen. At 48 h, unbound ^89^Zr-amatuximab would have crossed the tumor vasculature and diffused into tumor ECS (5.36 nM based on 7.74%ID/g tumor at 48 h and 53.6 nM in the ECS, assuming that ECS constitutes 10% of total tumor volume). Because the concentration of shed MSLN is ~300 nM in the ECS of a tumor of 300 mm^3^, ^89^Zr-amatuximab (53.6 nM) in the ECS would mostly exist as an antibody-Ag complex distributed throughout the entire tumor, presumably bypassing the binding sites on the surface of tumor cells nearest to the vasculature. On the other hand, ^89^Zr-amatuximab injected at 60 *µ*g dose results in an estimated blood concentration of 260 nM immediately after injection and 28.3 nM at 48 h (6.8%ID/g blood), that is, at a molar excess compared to the average concentration of shed MSLN in the blood (~6 nM). Consequently, ^89^Zr-amatuximab would remain mostly unbound, overcoming the sequestration into the hepatic reticuloendothelial system during a 48 h period. Therefore, a larger portion of the injected dose (95.2 nM based on 22.90%ID/g tumor at 48 h and 952 nM in the ECS) would have crossed the tumor vasculature and diffused into the tumor ECS. This concentration (952 nM) is larger than the shed MSLN concentration in the tumor ECS. The excess of ^89^Zr-amatuximab not bound to shed MSLN would bind to MSLN on tumor cells in the periphery rather than in the tumor core, as observed for ^89^Zr-B3 as well as Alexa-labeled B3 [24]. According to Boucher et al. the interstitial fluid pressure is often elevated in solid tumors but declines in the tumor periphery in the outer 0.2–1.1 mm [25]. Therefore, the accumulation in the tumor periphery might be favored by the lower interstitial fluid pressure in this region allowing for more antibody extravasation than in tumor core.

It is noteworthy that our PET study demonstrated that the tumor localized outside of the abdomen could be visualized even with the lower dose (10 *µ*g) at 24 h and thereafter because the tumor-to-blood and tumor-to-muscle ratios of ^89^Zr-amatuximab were higher than 2. However, it would be necessary to inject the higher dose (60 *µ*g) to visualize the tumor in the abdominal area. The PET study also suggests that ^89^Zr-amatuximab could be useful in a clinical setting because ^89^Zr with a long half-life (78.4 h) would be more suitable for the detection of tumor and the tumor-to-background ratio increased over time with the injection of the higher dose (60 *µ*g).

In this study, we demonstrated the effect of shed mesothelin on the tumor targeting and tumor microdistribution of anti-mesothelin mAb amatuximab in A431/H9 tumor using anti-Lewis-Y B3 as a negative control. However, it is possible that other shed antigens with a different binding epitope or a different affinity for the targeting mAb might show a different degree of shed antigen effects. Thus, careful investigation of other shed antigen systems would be needed to determine whether our findings could be generalized.

## 5. Conclusion

The use of A431/H9 tumor which overexpresses both the shed Ag mesothelin and the nonshed Ag Lewis-Y as a tumor model and the use of anti-mesothelin mAb amatuximab and anti-Lewis-Y mAb B3 as model mAbs for the BD, PET, and autoradiography studies allowed us to make a direct assessment on the effect of the shed Ag on the tumor and organ uptakes and tumor penetration, apart from the effects of other factors related to the tumor microenvironment. In addition, the use of ^89^Zr with a 3.4-day half-life to label the mAbs for PET imaging and autoradiography studies provided the advantages of high image sensitivity and resolution which enabled us to quantify the tumor and organ uptakes as well as tumor penetration of the mAbs.

The findings of this study imply that the systemic mAb concentration should be at a severalfold molar excess to the shed Ag concentration in the blood to increase the concentration of free mAb available for the tumor uptake and reduce the fraction of the mAb bound to the shed Ag in the blood and the subsequent hepatic processing of the mAb-Ag complexes. However, the mAb concentration in the tumor ECS should remain lower than the shed Ag concentration in the tumor ECS to maximize the tumor penetration of the mAb-Ag complexes by bypassing the binding site barrier. It will be the future challenge for both antibody engineers and clinical investigators to find this balance.

## Figures and Tables

**Table 1:** Effect of mAb dose on tumor-to-blood and tumor-to-organ uptake ratios of ^89^Zr-amatuximab (111 kBq/2, 10 or 60 *µ*g) or ^89^Zr-B3 (74 kBq/2, 15 or 60 *µ*g) in nude mice with A431/H9 tumor. All values are reported as mean ± standard deviation (*n* = 5).

mAb	Time	Injection dose	Tumor/blood	Tumor/liver	Tumor/kidney	Tumor/spleen	Tumor/muscle
Amatuximab	24 h	2 *µ*g	2.66 ± 0.43	0.11 ± 0.06	0.73 ± 0.32	0.23 ± 0.10	3.67 ± 0.86
Amatuximab	24 h	10 *µ*g	2.30 ± 1.35	0.24 ± 0.07	1.32 ± 0.49	0.38 ± 0.25	5.54 ± 2.00
Amatuximab	24 h	60 *µ*g	1.37 ± 0.54	0.66 ± 0.22	2.05 ± 0.54	1.01 ± 0.28	8.97 ± 2.44
B3	24 h	2 *µ*g	0.98 ± 0.34	1.00 ± 0.27	1.59 ± 0.39	2.71 ± 0.88	4.58 ± 1.23
B3	24 h	15 *µ*g	0.80 ± 0.17	0.89 ± 0.19	1.57 ± 0.22	1.95 ± 0.44	4.23 ± 0.81
B3	24 h	60 *µ*g	0.74 ± 0.10	0.90 ± 0.13	1.48 ± 0.18	2.15 ± 0.26	4.01 ± 0.57

**Table 2:** Effect of mAb dose on tumor-to-blood and tumor-to-organ uptake ratios of ^89^Zr-amatuximab (2.96 MBq/10 or 60 *µ*g) or ^89^Zr-B3 (2.22 MBq/15 or 60 *µ*g) in nude mice with A431/H9 tumor. The ratios were calculated by maximum uptake values from ROI analysis of PET images. Blood-H represents the blood activity in the heart. All values are reported as mean ± standard deviation (*n* = 5).

mAb	Time	Injection dose	Tumor/liver	Tumor/spleen	Tumor/muscle	Tumor/blood-H
Amatuximab	3 h	10 *µ*g	0.31 ± 0.06	0.65 ± 0.14	5.03 ± 1.53	0.38 ± 0.10
Amatuximab	24 h	10 *µ*g	0.40 ± 0.03	0.69 ± 0.21	10.20 ± 1.05	2.10 ± 0.64
Amatuximab	48 h	10 *µ*g	0.35 ± 0.02	0.54 ± 0.21	13.31 ± 1.68	3.21 ± 0.77
Amatuximab	3 h	60 *µ*g	0.36 ± 0.03	0.84 ± 0.18	9.57 ± 3.52	0.36 ± 0.03
Amatuximab	24 h	60 *µ*g	1.04 ± 0.07	1.98 ± 0.63	18.90 ± 1.57	1.68 ± 0.17
Amatuximab	48 h	60 *µ*g	1.02 ± 0.20	2.68 ± 0.86	31.16 ± 5.58	3.46 ± 0.69
B3	3 h	15 *µ*g	0.30 ± 0.03	0.89 ± 0.14	6.11 ± 0.45	0.35 ± 0.03
B3	24 h	15 *µ*g	0.72 ± 0.10	2.15 ± 0.29	14.26 ± 3.60	1.09 ± 0.15
B3	48 h	15 *µ*g	1.30 ± 0.01	3.67 ± 1.34	20.00 ± 4.08	2.15 ± 0.74
B3	3 h	60 *µ*g	0.28 ± 0.03	0.86 ± 0.19	5.51 ± 1.84	0.32 ± 0.02
B3	24 h	60 *µ*g	0.82 ± 0.21	2.40 ± 0.68	14.53 ± 5.49	1.24 ± 0.34
B3	48 h	60 *µ*g	1.21 ± 0.05	3.12 ± 0.35	19.03 ± 1.59	1.98 ± 0.15
